# Surveying Hematologists’ Perceptions and Readiness to Embrace Artificial Intelligence in Diagnosis and Treatment Decision-Making

**DOI:** 10.7759/cureus.49462

**Published:** 2023-11-26

**Authors:** Turki Alanzi, Fehaid Alanazi, Bushra Mashhour, Rahaf Altalhi, Atheer Alghamdi, Mohammed Al Shubbar, Saud Alamro, Muradi Alshammari, Lamyaa Almusmili, Lena Alanazi, Saleh Alzahrani, Raneem Alalouni, Nouf Alanzi, Ali Alsharifa

**Affiliations:** 1 Department of Health Information Management and Technology, College of Public Health, Imam Abdulrahman Bin Faisal University, Dammam, SAU; 2 Department of Clinical Laboratory Sciences, College of Applied Medical Sciences, Jouf University, Sakakah, SAU; 3 College of Pharmacy, ‏Jazan University, Jazan, SAU; 4 College of Pharmacy, Taif University, Taif, SAU; 5 College of Medicine, Imam Abdulrahman Bin Faisal University, Dammam, SAU; 6 College of Pharmacy, Northern Border University, Arar, SAU; 7 College of Medicine, King Abdulaziz University, Rabigh, SAU; 8 College of Public Health, Imam Abdulrahman Bin Faisal University, Dammam, SAU; 9 College of Medicine, ‏Cairo University, Cairo, EGY

**Keywords:** decision-making, ai adoption, ai awareness, hematologists, hematology, artificial intelligence

## Abstract

Aim: This study aims to explore the critical dimension of assessing the perceptions and readiness of hematologists to embrace artificial intelligence (AI) technologies in their diagnostic and treatment decision-making processes.

Methods: This study used a cross-sectional design for collecting data related to the perceptions and readiness of hematologists using a validated online questionnaire-based survey. Both hematologists (MD) and postgraduate MD students in hematology were included in the study. A total of 188 participants, including 35 hematologists (MD) and 153 MD hematology students, completed the survey.

Results: Major challenges include "AI's level of autonomy" and "the complexity in the field of medicine." Major barriers and risks identified include "lack of trust," "management's level of understanding," "dehumanization of healthcare," and "reduction in physicians' skills." Statistically significant differences in perceptions of benefits including resources (p=0.0326, p<0.05) and knowledge (p=0.0262, p<0.05) were observed between genders. Older physicians were observed to be more concerned about the use of AI compared to younger physicians (p<0.05).

Conclusion: While AI use in hematology diagnosis and treatment decision-making is positively perceived, issues such as lack of trust, transparency, regulations, and poor AI awareness can affect the adoption of AI.

## Introduction

Artificial intelligence (AI) technologies are applications or machines designed "to perform the cognitive functions we associate with human minds, such as perceiving, reasoning, learning, interacting with an environment, problem-solving, and even exercising creativity" [1]. In recent years, the healthcare landscape has witnessed a transformative shift with the integration of AI technologies into various medical specialties such as healthcare assistance, diagnosis, remote monitoring, and decision-making. This paradigm shift has not only revolutionized diagnostic accuracy and treatment efficacy but has also posed unique challenges to healthcare professionals in adapting to these advanced tools [2,3]. In the realm of hematology, where precise and timely diagnosis is paramount, the assimilation of AI holds immense potential to enhance patient care outcomes. Technologies have ushered in a new era of possibilities for medical professionals, particularly hematologists, by offering advanced tools that can significantly enhance their diagnostic accuracy, treatment decision-making, and overall patient care [2,3].

In the realm of hematology, where precision and timely interventions are crucial, AI's potential benefits are particularly profound [4]. First and foremost, AI technologies can augment hematologists’ diagnostic capabilities. Hematological disorders often involve complex patterns and subtle nuances that can be challenging for even experienced clinicians to identify [5]. AI-powered algorithms excel at analyzing vast amounts of data, such as blood cell morphology, genetic profiles, and biomarker levels, to detect patterns that might escape human observation. This can lead to quicker and more accurate diagnoses of conditions like anemia, leukemia, and clotting disorders, ultimately leading to better patient outcomes [6-8]. Additionally, AI can facilitate personalized medicine in hematology. Each patient's physiology and genetic makeup are unique, influencing how they respond to treatments. AI can assimilate data from a patient's medical history, genetic information, and even lifestyle factors to assist hematologists in tailoring treatment plans that are optimized for individual patients. This level of personalization can result in more effective treatments, minimized side effects, and faster recovery times [6].

AI also streamlines the process of treatment decision-making. Hematologists often face intricate choices when determining the most suitable treatment regimen for their patients. AI can analyze a plethora of clinical guidelines, research papers, and patient records to present evidence-based recommendations, enabling hematologists to make well-informed decisions efficiently [9]. This not only saves valuable time but also reduces the risk of human error in the decision-making process [10,11]. In the domain of research, AI can accelerate advancements in hematology. By analyzing vast datasets from clinical trials, genetics studies, and molecular research, AI can identify potential targets for new treatments and therapies. Hematologists can leverage these insights to contribute to the development of innovative treatments for various blood-related disorders, fostering progress in the field.

Moreover, AI technologies can aid in monitoring patient progress [12]. Continuous monitoring of hematological parameters is vital for conditions like anemia, hemophilia, and bone marrow disorders [13]. AI-powered monitoring systems can track changes in blood counts and other relevant markers, alerting hematologists to any deviations from the expected course. This early detection allows for timely interventions, preventing complications and ensuring patients receive optimal care [14,15]. However, the integration of AI in hematology also comes with challenges. Hematologists need to be well-versed in AI concepts to effectively collaborate with technologists and data scientists in developing and refining these tools. AI training programs are being introduced for healthcare workers in order to prepare them for future AI-enabled healthcare services [16]. Furthermore, ethical concerns regarding patient data privacy, the transparency of AI algorithms, and the potential bias in AI models must be addressed to ensure the responsible and ethical use of these technologies [17,18].

Analyzing the perceptions and readiness of hematologists to adopt AI technologies for diagnosis and treatment decision-making is of paramount importance in the field of hematology. Hematologists play a crucial role in diagnosing and treating blood-related disorders, where timely and accurate decisions are often a matter of life and death. AI technologies have the potential to revolutionize this field by assisting hematologists in processing and interpreting vast amounts of patient data efficiently and with high precision. This can lead to quicker and more accurate diagnoses, personalized treatment plans, and improved patient outcomes. However, the successful integration of AI into clinical practice hinges on the willingness and readiness of hematologists to embrace these technologies, understand their capabilities, and trust their recommendations. Therefore, studying hematologists' perceptions and readiness is essential for ensuring the effective adoption of AI tools and the continuous enhancement of patient care in hematology. Accordingly, this study aims to explore the critical dimension of assessing the perceptions and readiness of hematologists to embrace AI technologies in their diagnostic and treatment decision-making processes.

## Materials and methods

Study setting and participants

This study used a cross-sectional design to collect data related to the perceptions and readiness of hematologists to embrace AI technologies in diagnosis and treatment decision-making in Saudi Arabia from September 07, 2023, to October 17, 2023. The research took into account both hematologists (MD) and postgraduate MD students in hematology. Initially, eleven hospitals and affiliated educational institutions across Saudi Arabia were considered for recruiting participants by sending participation request emails. The strategy chosen was the use of an online survey questionnaire to make it easier for nurses to openly voice their ideas while also increasing their level of comfort.

Recruitment and sampling

As the participants were purposively recruited from the selected hospitals, convenience and purposive sampling techniques were adopted [19]. Additionally, the snowball sampling technique [20] was used for hematologists (MD), with requests made for them to forward the survey link to their colleagues due to the relatively low number of hematologists in the selected hospitals. However, MD hematology students were only selected from the eleven hospitals, and no snowball sampling technique was applied to them.

Instruments

To collect information, a questionnaire survey (Appendices section) was built using Google Forms (Google LLC, California, USA) and distributed online through emails. The survey questionnaire is divided into two sections. The first section focuses on collecting demographic information related to age, gender, and role. The second section focuses on gathering information about the readiness of AI implementation in hematology. A total of nine questions were adopted from various studies. Questions related to strategic alignment (four items), resources (three items), knowledge (three items), and culture (three items) were adopted from Johnk et al. [21]. Additionally, other factors, including training, reasons to attend training (five items), challenges (six items), barriers (five items), and risks (four items) of implementing AI in hematology, were adopted from Boillat et al. [22]. Initially, the questionnaire underwent a review by two professors from the hematology department at Imam Abdulrahman Bin Faisal University, who suggested and incorporated a few changes in the statement formations. Later, a pilot study was conducted with nine MD hematology students and three hematologists (MD), and the data were analyzed. Cronbach alpha was calculated for all items and was observed to be greater than 0.7, indicating good internal consistency [23].

Ethical considerations

All the participants were fully informed about the study through an information sheet attached to the invitation email. Informed consent was obtained from all the participants using a check button before starting the survey. Participation was voluntary, and participants were assured of their anonymity and their rights with respect to the data. Ethical approval was received from the ethics committee of Imam Abdulrahman Bin Faisal University (IRB-2023-03-327).

Data collection

A participant information sheet was attached along with the invitation email (containing the survey link), explaining the rights of the participants, and was forwarded to the respective institutions. The email was further forwarded by the institutions to the hematologists (MD) and students in MD hematology at the respective hospitals. At the end of four weeks, a total of 188 individuals participated in the study, including 81 hematologists (MD) and 107 MD hematology students.

Data analysis

To attain the objectives of the research, the researcher utilized SPSS Statistics version 24 (IBM Corp. Released 2016. IBM SPSS Statistics for Windows, Version 24.0. Armonk, NY: IBM Corp.) to analyze the data. Descriptive statistics were used to characterize the participants’ demographic data. Additionally, two-sample t-tests with unequal variances and a single-factor ANOVA were employed to analyze the data.

## Results

The participants' demographic information is presented in Table 1. About 68.6% are male and 31.4% are female. The majority of the participants are in the age group of 18-30 years (50.5%), followed by 31-40 years (41%), and 41-50 years (8.5%). Approximately 18.6% of the participants are hematologists (MD), and 81.4% are MD students in hematology.

**Table 1:** Participants' demographics

		N	Relative frequency
Gender	Male	129	68.6%
	Female	59	31.4%
Age (in years)	18-30	95	50.5%
	31-40	77	41.0%
	41-50	16	8.5%
Role	Hematologist (MD)	35	18.6%
	MD hematologist student	153	81.4%

As shown in Table 2, the main benefit of the AI training course, as perceived by the participants, is to better understand the concepts of AI and the opportunities it offers in hematology.

**Table 2:** Benefits of attending an AI-training course

Benefits	Mean
Better understand the main concept of AI	3.98
Explore new opportunities offered by AI in general	2.93
Explore new opportunities offered by AI in medicine and in my field	3.97
Know more about existing commercial solutions	3.59
Create my own AI algorithms or applications	2.36

Although the benefits are realized among participants, nearly 32.4% have never attended any training course. Interestingly, 46.8% of the participants attended a course in 2023, 13.8% in 2022, and 6.9% prior to 2020. Table 3 presents the mean scores for a range of challenges, barriers, and risks associated with the implementation of AI for diagnosis and treatment decision-making in the field of medicine. These mean scores provide valuable insights into the perceived significance of each issue within the context of AI integration in healthcare.

**Table 3:** Challenges, barriers, and risks in implementation of AI for diagnosis and treatment decision-making

		Mean
Challenges	Outcomes of AI algorithms are difficult to trace or understand (the black-box syndrome)	3.15
	The complexity of the field of medicine	3.51
	The availability of high-quality data samples	2.89
	The AI's level of autonomy (what AI should and should not do)	4.25
	The costs associated with the implementation of AI	2.95
	Data privacy/confidentiality	2.84
Barriers	The availability of comparison studies	2.96
	The safe use of AI	2.91
	Build trust between humans and AI	3.75
	Availability of regulations and legislation	2.95
	The top management's level of understanding	3.99
Risks	Dehumanization of healthcare	2.89
	Reduction in physicians' skills (e.g., hematologists might execute fewer types of tasks)	2.82
	AI will eventually harm patients	2.85
	Hematologists may become redundant	2.71

In terms of challenges, two notable concerns stand out. Firstly, there is the challenge of AI algorithms' outcomes being difficult to trace or understand, often referred to as the "black-box syndrome," with a mean score of 3.15. This suggests that healthcare professionals may find it challenging to trust AI-driven decisions when they cannot easily interpret the rationale behind those decisions. Secondly, the complexity of the field of medicine is rated at 3.51, indicating that the intricacies of medical practice present a significant hurdle for AI adoption. This complexity underscores the need for AI systems that can navigate and understand the intricacies of healthcare. Moving on to barriers, building trust between humans and AI (mean score: 3.75) and the top management's level of understanding (mean score: 3.99) emerge as crucial factors. Building trust is essential to gaining the acceptance of AI in healthcare, while the management's understanding and support can greatly influence the successful adoption of AI technologies. Additionally, the availability of comparison studies (mean score: 2.96) and ensuring the safe use of AI (mean score: 2.91) are identified as important barriers to address. The perceived risks related to AI implementation in healthcare are characterized by concerns such as the dehumanization of healthcare (mean score: 2.89), the potential reduction in physicians' skills (mean score: 2.82), the fear that AI could harm patients (mean score: 2.85), and the possibility of healthcare professionals, such as hematologists, becoming redundant (mean score: 2.71). These risks highlight the need for careful planning and ethical considerations when integrating AI into medical practice, as well as the importance of maintaining the essential human touch in healthcare delivery.

Table 4 provides insightful data regarding the benefits of implementing AI in the context of diagnosis and treatment decision-making for hematologists, as well as their corresponding mean scores. These results shed light on the perceived advantages and facilitating factors that can enhance the integration of AI in hematology practice. Under the category of "strategic alignment," several key factors stand out. "AI business potentials," with a mean score of 3.58, underscore the versatility and broad applicability of AI in hematology, suggesting that it can be leveraged in various ways to benefit the field. "Stakeholders AI readiness," rated at 4.02, emphasizes the importance of preparedness among both internal and external stakeholders to effectively utilize AI-integrated offerings. "Top management support," with a mean score of 3.26, signifies the role of leadership in signaling the strategic significance of AI and fostering initiatives. Lastly, "AI-process fit," rated at 3.56, suggests that standardization and process reengineering can facilitate the seamless integration of AI into hematology practices.

**Table 4:** Benefits of AI for diagnosis and treatment decision-making for hematologists

		Mean
Strategic alignment	AI business potentials: AI functions are highly versatile and broadly applicable in hematology	3.58
	Stakeholders AI readiness: AI readiness enables internal or external customers to appropriately use AI-integrated offerings	4.02
	Top management Support: op management support signals AI’s strategic relevance to the organization and fosters AI initiatives	3.26
	AI-process fit: AI-process fit through standardization, reengineering, and implementation of new processes facilitates AI adoption	3.56
Resources	Financial budget: strategic allocation of the financial budget for AI adoption supports the overcoming of initial obstacles and uncertainty	3.94
	Personnel: AI specialists and business analysts with AI know-how facilitate AI adoption	2.94
	IT infrastructure: IT infrastructure enables AI-related activities and AI integration	3.91
Knowledge	AI awareness: AI awareness ensures that hematologists have an adequate understanding and expectations of AI	3.06
	Upskilling: enables hematologists to learn and develop AI or AI-related skills	3.89
	AI ethics: AI ethics comprise measures to prevent bias, safety violations, or discrimination in AI outcomes	4.07
Culture	Innovativeness: increases hematologists’ willingness to change the status quo through the application of AI	3.03
	Collaborative work: enables physicians to work in teams and combine different skills	3.07
	Change management: helps physicians understand and cope with AI-induced organizational change	3.23

In terms of "resources," financial allocation to AI adoption (mean score: 3.94) is seen as a critical factor in overcoming initial obstacles and uncertainties. Adequate personnel with AI expertise (mean score: 2.94) and the availability of a suitable IT infrastructure (mean score: 3.91) are essential to support AI-related activities and integration. Regarding "knowledge," "AI awareness" (mean score: 3.06) and "upskilling" (mean score: 3.89) are important elements for hematologists to understand and effectively work with AI. "AI ethics" (mean score: 4.07) is particularly noteworthy, indicating the significance of measures to prevent bias, safety violations, or discrimination in AI outcomes, a crucial aspect of AI integration in healthcare.

Within the "culture" category, "innovativeness" (mean score: 3.03) suggests that a culture of openness to change and innovation is essential for hematologists to embrace AI. "Collaborative work" (mean score: 3.07) highlights the benefits of teamwork and the combination of diverse skills, which can be facilitated by AI. "Change management" (mean score: 3.23) underscores the importance of helping physicians understand and adapt to the organizational changes driven by AI implementation.

Table 5 provides an analysis of differences in participants' perceptions of challenges, barriers, and risks across different groups based on gender and professional background (hematologist MD vs. MD hematologist student). There was no statistically significant difference in perceptions of challenges and risks between male and female participants. There was a marginally significant difference in perceptions of barriers (p=0.08), with males perceiving slightly more barriers than females. There was no statistically significant difference in perceptions of challenges, barriers, and risks between hematologists (MD) and MD hematologist students. However, there was a marginally significant difference in perceptions of risks (p=0.08), with MD hematologist students perceiving slightly more risks than hematologists (MD).

**Table 5:** Differences between the participants’ perceptions of challenges, barriers, and risks SD: standard deviation; df: degrees of freedom

	Variables	N	Mean	SD	df	t-value	p-value
Challenges	Male	129	3.3	0.37	88	0.87894	0.3815
	Female	59	3.21	0.49			
	Hematologist (MD)	35	3.39	0.45	49	1.17828	0.2443
	MD hematologist student	153	3.24	0.39			
Barriers	Male	129	3.38	0.39	98	1.8779	0.0607
	Female	59	3.17	0.55			
	Hematologist (MD)	35	3.38	0.56	47	0.60307	0.5493
	MD hematologist student	153	3.3	0.43			
Risks	Male	129	2.86	1.12	120	0.64387	0.5208
	Female	59	2.75	0.98			
	Hematologist (MD)	35	2.67	1.03	50	1.36137	0.0897
	MD hematologist student	153	2.87	1.06			

Table 6 provides an analysis of differences in participants' perceptions of the benefits of AI across different groups based on gender and professional background (hematologist MD vs. MD hematologist student). Statistically significant differences exist in perceptions of resources (p=0.0326, p<0.05) and knowledge (p=0.0262, p<0.05) based on gender, with males perceiving these aspects more positively than females. However, there are no statistically significant differences in perceptions of strategic alignment or culture based on gender, and there are no statistically significant differences in any of these aspects between hematologists (MD) and MD hematologist students.

**Table 6:** Differences between the participants’ perceptions of the benefits of AI * Statistically significant difference; SD: standard deviation; df: degrees of freedom

	Variables	N	Mean	SD	df	t-value	p-value
Strategic alignment	Male	129	3.65	0.74	100	1.04859	0.2968
	Female	59	3.5	0.98			
	Hematologist (MD)	35	3.6	1.04	46	0.27853	0.3909
	MD hematologist student	153	3.59	0.77			
Resources	Male	129	3.68	0.64	99	1.86381	0.0326*
	Female	59	3.41	0.86			
	Hematologist (MD)	35	3.71	0.96	47	0.80161	0.2134
	MD hematologist student	153	3.57	0.67			
Knowledge	Male	129	3.77	0.59	99	1.96553	0.0262*
	Female	59	3.46	1.11			
	Hematologist (MD)	35	3.78	0.87	45	075101	0.2281
	MD hematologist student	153	3.65	0.75			
Culture	Male	129	3.06	1.05	112	0.9792	0.1647
	Female	59	3.22	1.06			
	Hematologist (MD)	35	2.99	1.08	50	0.76627	0.2235
	MD hematologist student	153	3.14	1.05			

Table 7 presents the results of the ANOVA for the three age groups (18-30, 31-40, and 41-50) in relation to various factors, including strategic alignment, resources, knowledge, culture, challenges, barriers, and risks.

**Table 7:** ANOVA results for the three age groups (18-30, 31-40, and 41-50) in relation to challenges, barriers, risks, and benefits SS: sum of squares; df: degrees of freedom

	SS	df	MS	F	p-value	F crit	SS
Strategic alignment	0.593338	2	0.296669	0.360099	0.698095	3.044771	0.593338
Resources	2.020479	2	1.010239	1.403198	0.248413	3.044771	2.020479
Knowledge	0.463116	2	0.231558	0.297328	0.743155	3.044771	0.463116
Culture	1.557562	2	0.778781	0.735128	0.480839	3.044771	1.557562
Challenges	6.426658	2	3.213329	7.945113	0.000489*	3.044771	6.426658
Barriers	3.894397	2	1.947198	4.166308	0.016989*	3.044771	3.894397
Risks	21.4866	2	10.7433	10.61172	<0.0001*	3.044771	21.4866

There were no statistically significant differences in perceptions of any of the factors (strategic alignment, resources, knowledge, culture, challenges, barriers, and risks) among the three age groups (18-30, 31-40, and 41-50). This suggests that, in the context of the study, age did not appear to be a significant factor influencing how individuals perceived these aspects of their work or environment.

## Discussion

This study aims to analyze the perceptions and readiness of hematologists to embrace AI in diagnosis and treatment decision-making. Among the major challenges, AI's level of autonomy and the complexity in the field of medicine are identified as significant hurdles. Similarly, focusing on the barriers, a lack of trust and management's level of understanding are recognized as substantial obstacles. In relation to risks, dehumanization of healthcare and a reduction in physicians' skills are identified as major concerns. Although AI's level of autonomy is observed as one of the challenges [24-26], emphasis is placed on physicians' and patients' levels of autonomy in the ongoing research [27-30], indicating gaps in assessing AI's autonomy levels in hematologists' diagnosis and treatment decision-making. Barriers and risks such as trust issues, dehumanization, and reduction in physicians' skills have been observed in similar studies [31-34], but a lack of management understanding of AI is not highly observed in research studies. This indicates the significance of AI literacy among management and all healthcare workers [16] for preparedness for AI use in hematology diagnosis and treatment decision-making. Other issues like data protection, lack of transparency in clinical decision-making, and erosion of the doctor-patient relationship [35] are identified as a few issues in adopting AI in hematology.

The analysis of challenges, barriers, and risks indicates that there are some differences in perceptions among the groups, particularly in the case of barriers, where males tend to perceive slightly higher barriers than females, but these differences are not statistically significant. Additionally, the differences between hematologist MDs and MD hematologist students are not significant. The results suggest that while there are challenges, barriers, and risks associated with implementing AI in healthcare, there is also recognition of the potential benefits. However, clear regulations, building trust, and addressing safety concerns are key issues that need to be addressed.

These findings highlight the multifaceted nature of the benefits associated with AI adoption in hematology. They emphasize the importance of not only technical aspects but also strategic alignment, resource allocation, knowledge dissemination, and a supportive cultural environment in realizing the potential advantages of AI in diagnosis and treatment decision-making within the field. The analysis of benefits suggests that there are some differences in perceptions of benefits between genders and, to a certain extent, between professional categories, particularly in the case of resources and knowledge, where males perceive significantly higher benefits than females. However, in most dimensions, these differences are not statistically significant. Similarly, studies have identified no significant impact of gender on AI acceptance, indicating a high preference for AI use among both genders [36,37]. Furthermore, both hematologists (MD) and MD hematology students did not differ in their perception of the benefits of AI, indicating awareness of the importance of AI in their field and their level of understanding of AI. A recent study observed that many doctors were aware of the roles of AI in medicine but were not fully ready to adopt them [38]. This trend suggests that concerns about the lack of regulation and about accountability hinder acceptance. We are currently on the verge of a new phase characterized by the increased incorporation of AI into regular hematology procedures. This development holds the potential to significantly enhance patient care. However, in order to effectively utilize the advantages offered by AI, upcoming hematologists will require tailored educational opportunities that involve collaboration with data scientists [34].

The ANOVA results indicate that age has no statistically significant influence on how individuals perceive the benefits related to strategic alignment, resources, and knowledge in the context of AI implementation. However, significant differences were observed among the older age group (41-50 years) in relation to challenges, barriers, and risks compared to the younger age group (<41 years). These findings indicate that older participants perceive challenges, barriers, and risks to be more influential on AI use in hematology diagnosis and treatment compared to younger participants. Lack of AI awareness among older physicians could be a barrier to negative perceptions of AI use [39]. However, in a recent study, older physicians were also more positive about using clinical AI, indicating the importance of AI awareness as a significant factor in promoting AI use among physicians [38]. These findings suggest that when addressing AI-related concerns and communication, it may be beneficial to consider age-related differences, especially in the areas of challenges, barriers, and risks.

Based on these findings, both practical and theoretical implications can be drawn. This study shows that some MD hematology students perceive that AI could improve healthcare outcomes in the context of hematology. Training and education are immediate practical ramifications of the study. The findings suggest that hematologists and other healthcare professionals must understand AI ideas and capabilities to work with engineers and data scientists to develop and improve AI solutions. This highlights the necessity for continual education and training to prepare healthcare workers for AI-enabled services. Programs could enable healthcare workers to connect their medical skills with AI technology. Ethics is another important practical issue with AI adoption. The study raises issues about patient data privacy, AI algorithm openness, and AI model bias. To deploy AI technology ethically in healthcare, policymakers, healthcare institutions, and AI developers must address these ethical issues. The need for strong ethical frameworks, rules, and guidelines for AI in healthcare is clear to protect patients' rights and assure fair and unbiased AI-driven decision-making. The report also emphasizes management support for AI adoption. Top management's involvement in AI efforts can demonstrate AI's strategic importance and offer the resources and leadership needed for success. Leadership in healthcare should actively design and execute AI integration plans. Theory-wise, the work adds to the literature on AI use in healthcare, notably in hematology. It illuminates the unique perceptions, obstacles, and preparedness of hematologists to utilize AI. This research enhances the theoretical understanding of how AI technology might be implemented in specialized medical practices and exposes its challenges and prospects.

Despite its valuable insights and contributions, this study has several limitations that warrant acknowledgment. Firstly, the study's sample size, while representative of hematologists in Saudi Arabia, may not fully capture the diversity of perspectives and readiness levels among hematologists globally. Secondly, the study's cross-sectional design provides a snapshot of participants' perceptions and readiness at a specific point in time. Longitudinal research could offer a more comprehensive understanding of how these perceptions evolve over time, especially as AI technologies continue to advance and become more integrated into healthcare. Moreover, while the study identifies challenges, barriers, and risks associated with AI adoption in hematology, it does not delve deeply into potential solutions or strategies to address these issues. Future research could focus on developing and evaluating interventions aimed at mitigating the identified challenges and facilitating the responsible integration of AI in hematology practice.

## Conclusions

This study examines Saudi hematologists’ views on AI. Most participants expected AI to improve diagnosis and treatment decisions. They envisioned higher diagnosis accuracy, personalized treatment, faster decision-making, and expedited hematology research. Such benefits could enhance patient outcomes and healthcare efficiency. However, this study highlights key obstacles, restrictions, and perceived hazards of AI adoption in hematology. The intricacy of medical practice and the "black-box syndrome," where AI outcomes become impossible to trace, are identified as issues. Building trust with AI and garnering senior management support pose challenges. Potential risks include healthcare dehumanization, deterioration in medical skills, and AI causing harm. The study concludes that healthcare practitioners, especially hematologists, require AI literacy and must address data protection, AI algorithm openness, and ethics. Ethical AI use in hematology requires specific regulations and norms, according to the study. It stresses the importance of monitoring healthcare practitioners' AI attitudes and readiness, especially in hematology, where quick and accurate decisions can save lives. Understanding and overcoming these issues are essential for adopting AI into medical practice and improving patient care. Healthcare organizations, policymakers, and educational institutions must collaborate to provide comprehensive training, create AI-aware cultures, and establish ethical AI use norms in hematology and across healthcare. AI has the potential to disrupt healthcare while meeting the highest patient care and safety standards.

## Appendices

Survey questionnaire

Demographics:

1.      Age: 18-30/31-40/41-50/>50

2.      Gender: male/female

3.      Role: Hematologist (MD)/MD Hematologist student

Main questions

1.      In relation to your unit/based on your perception, please rate your level of agreeableness on the following factors related to "strategic alignment" on a scale of 1 to 5 (1: strongly disagree; 2: disagree; 3: neutral; 4: agree; 5: strongly agree)

·         AI business potentials: AI functions are highly versatile and broadly applicable in hematology

·         Stakeholders AI readiness: AI readiness enables internal or external customers to appropriately use AI-integrated offerings

·         Top management support: op management support signals AI’s strategic relevance to the organization and fosters AI initiatives

·         AI-process fit: AI-process fit through standardization, reengineering, and implementation of new processes facilitates AI adoption

2.      In relation to your unit/based on your perception, please rate your level of agreeableness on the following factors related to "resources" on a scale of 1 to 5 (1: strongly disagree; 2: disagree; 3: neutral; 4: agree; 5: strongly agree)

·         Financial budget: strategic allocation of the financial budget for AI adoption supports the overcoming of initial obstacles and uncertainty

·         Personnel: AI specialists and business analysts with AI know-how facilitate AI adoption

·         IT infrastructure: IT infrastructure enables AI-related activities and AI integration

3.      In relation to your unit/based on your perception, please rate your level of agreeableness on the following factors related to "knowledge" on a scale of 1 to 5 (1: strongly disagree; 2: disagree; 3: neutral; 4: agree; 5: strongly agree)

·         AI awareness: AI awareness ensures that hematologists have adequate understanding and expectations of AI

·         Upskilling: enables hematologists to learn and develop AI or AI-related skills

·         AI ethics: AI ethics comprise measures to prevent bias, safety violations, or discrimination in AI outcomes

4.      Have you attended a course on artificial intelligence?

·         Never

·         This year

·         Last year

·         Two to three years ago

·         More than three years ago

5.      Would you benefit (reasons to attend a course or training on AI) more from training to (1: strongly disagree; 2: disagree; 3: neutral; 4: agree; 5: strongly agree)

·         Better understand the main concept of AI

·         Explore new opportunities offered by AI in general

·         Explore new opportunities offered by AI in medicine and in my field

·         Know more about existing commercial solutions

·         Create my own AI algorithms or applications

6.      Please rate the following challenges of implementing AI technologies in hematology on a scale of 1 to 5 (1: strongly disagree; 2: disagree; 3: neutral; 4: agree; 5: strongly agree)

·         Outcomes of AI algorithms are difficult to trace or understand (the black-box syndrome)

·         The complexity of the field of medicine

·         The availability of high-quality data samples

·         The AI's level of autonomy (what AI should and should not do)

·         The costs associated with the implementation of AI

·         Data privacy/confidentiality

7.      Please rate the following barriers to the implementation of AI technologies in hematology on a scale of 1 to 5 (1: strongly disagree; 2: disagree; 3: neutral; 4: agree; 5: strongly agree)

·         The availability of comparison studies

·         The safe use of AI

·         Build trust between humans and AI

·         Availability of regulations and legislation

·         The top management's level of understanding

8.      Please rate the following risks to the implementation of AI technologies in hematology on a scale of 1 to 5 (1: strongly disagree; 2: disagree; 3: neutral; 4: agree; 5: strongly agree)

·         Dehumanization of healthcare

·         Reduction in physicians' skills (e.g., hematologists might execute fewer types of tasks)

·         AI will eventually harm patients

·         Hematologists may become redundant

9.      In relation to your unit/based on your perception, please rate your level of agreeableness on the following factors related to "culture" on a scale of 1 to 5 (1: strongly disagree; 2: disagree; 3: neutral; 4: agree; 5: strongly agree)

·         Innovativeness: increases hematologists' willingness to change the status quo through the application of AI

·         Collaborative work: enables employees to work in teams and combine different skills

·         Change management: helps employees understand and cope with AI-induced organizational change

## References

[1] (2023). What is AI?. https://www.mckinsey.com/featured-insights/mckinsey-explainers/what-is-ai#.

[2] Kumar Y, Koul A, Singla R, Ijaz MF (2023). Artificial intelligence in disease diagnosis: a systematic literature review, synthesizing framework and future research agenda. J Ambient Intell Humaniz Comput.

[3] Chen JH, Dhaliwal G, Yang D (2022). Decoding artificial intelligence to achieve diagnostic excellence: learning from experts, examples, and experience. JAMA.

[4] Coutsouvelis J, Corallo CE, Dooley MJ, Foo J, Whitfield A (2010). Implementation of a pharmacist-initiated pharmaceutical handover for oncology and haematology patients being transferred to critical care units. Support Care Cancer.

[5] Valent P, Orfao A, Kubicek S (2021). Precision medicine in Hematology 2021: definitions, tools, perspectives, and open questions. Hemasphere.

[6] Radakovich N, Nagy M, Nazha A (2020). Artificial intelligence in hematology: current challenges and opportunities. Curr Hematol Malig Rep.

[7] Kaestner L (2020). Artificial intelligence meets hematology. Transfus Apher Sci.

[8] El Alaoui Y, Elomri A, Qaraqe M (2022). A review of artificial intelligence applications in hematology management: current practices and future prospects. J Med Internet Res.

[9] Busnatu Ș, Niculescu AG, Bolocan A (2022). Clinical applications of artificial intelligence-an updated overview. J Clin Med.

[10] Walter W, Haferlach C, Nadarajah N, Schmidts I, Kühn C, Kern W, Haferlach T (2021). How artificial intelligence might disrupt diagnostics in hematology in the near future. Oncogene.

[11] Passamonti F, Corrao G, Castellani G, Mora B, Maggioni G, Gale RP, Della Porta MG (2022). The future of research in hematology: integration of conventional studies with real-world data and artificial intelligence. Blood Rev.

[12] Guo J, Li B (2018). The application of medical artificial intelligence technology in rural areas of developing countries. Health Equity.

[13] (2023). Techniques for hematological disorders. Advances in the diagnosis and evaluation of disabling physical health conditions.

[14] Walter W, Pohlkamp C, Meggendorfer M, Nadarajah N, Kern W, Haferlach C, Haferlach T (2023). Artificial intelligence in hematological diagnostics: game changer or gadget?. Blood Rev.

[15] Rösler W, Altenbuchinger M, Baeßler B (2023). An overview and a roadmap for artificial intelligence in hematology and oncology. J Cancer Res Clin Oncol.

[16] Charow R, Jeyakumar T, Younus S (2021). Artificial intelligence education programs for health care professionals: scoping review. JMIR Med Educ.

[17] Bartoletti I (2019). Ai in healthcare: ethical and privacy challenges. Artificial intelligence in medicine.

[18] Gedefaw L, Liu CF, Ip RK, Tse HF, Yeung MH, Yip SP, Huang CL (2023). Artificial intelligence-assisted diagnostic cytology and genomic testing for hematologic disorders. Cells.

[19] Etikan I (2016). Comparison of convenience sampling and purposive sampling. Am J Theor Appl Stat.

[20] Kirchherr J, Charles K (2018). Enhancing the sample diversity of snowball samples: recommendations from a research project on anti-dam movements in Southeast Asia. PLoS One.

[21] Jöhnk J, Weißert M, Wyrtki K (2020). Ready or not, AI comes— an interview study of organizational AI readiness factors. Bus Inf Syst Eng.

[22] Boillat T, Nawaz FA, Rivas H (2022). Readiness to embrace artificial intelligence among medical doctors and students: questionnaire-based study. JMIR Med Educ.

[23] Taber KS (2018). The use of Cronbach’s alpha when developing and reporting research instruments in science education. Res Sci Educ.

[24] Farhud DD, Zokaei S (2021). Ethical issues of artificial intelligence in medicine and healthcare. Iran J Public Health.

[25] Szklanna PB, Weiss L, Namee BM, Faryal R, Kevane B, Ní Áinle F, Maguire PB (2023). Ai in haematology. AI in clinical medicine: a practical guide for healthcare professionals.

[26] Tiribelli S (2023). The AI ethics principle of autonomy in health recommender systems. Argumenta.

[27] Amann J, Blasimme A, Vayena E, Frey D, Madai VI (2020). Explainability for artificial intelligence in healthcare: a multidisciplinary perspective. BMC Med Inform Decis Mak.

[28] Dalton-Brown S (2020). The ethics of medical AI and the physician-patient relationship. Camb Q Healthc Ethics.

[29] Reddy S, Allan S, Coghlan S, Cooper P (2020). A governance model for the application of AI in health care. J Am Med Inform Assoc.

[30] Di Nucci E (2019). Should we be afraid of medical AI?. J Med Ethics.

[31] Bohr A, Memarzadeh K (2020). The rise of artificial intelligence in healthcare applications. Artificial Intelligence in Healthcare.

[32] Formosa P, Rogers W, Griep Y, Bankins S, Richards D (2022). Medical AI and human dignity: contrasting perceptions of human and artificially intelligent (AI) decision making in diagnostic and medical resource allocation contexts. Comput Human Behav.

[33] Asan O, Bayrak AE, Choudhury A (2020). Artificial intelligence and human trust in healthcare: focus on clinicians. J Med Internet Res.

[34] Chen M, Zhang B, Cai Z (2022). Acceptance of clinical artificial intelligence among physicians and medical students: a systematic review with cross-sectional survey. Front Med (Lausanne).

[35] Chai SY, Hayat A, Flaherty GT (2022). Integrating artificial intelligence into haematology training and practice: opportunities, threats and proposed solutions. Br J Haematol.

[36] Al-Medfa MK, Al-Ansari AM, Darwish AH, Qreeballa TA, Jahrami H (2023). Physicians' attitudes and knowledge toward artificial intelligence in medicine: benefits and drawbacks. Heliyon.

[37] Chalutz Ben-Gal H (2022). Artificial intelligence (AI) acceptance in primary care during the coronavirus pandemic: what is the role of patients' gender, age and health awareness? A two-phase pilot study. Front Public Health.

[38] Tamori H, Yamashina H, Mukai M, Morii Y, Suzuki T, Ogasawara K (2022). Acceptance of the use of artificial intelligence in medicine among Japan’s doctors and the public: a questionnaire survey. JMIR Hum Factors.

[39] AlZaabi A, AlMaskari S, AalAbdulsalam A (2023). Are physicians and medical students ready for artificial intelligence applications in healthcare?. Digit Health.

